# Distinguishing Benign from Malignant Pancreatic and Periampullary Lesions Using Combined Use of ^1^H-NMR Spectroscopy and Gas Chromatography–Mass Spectrometry

**DOI:** 10.3390/metabo7010003

**Published:** 2017-01-13

**Authors:** Yarrow J. McConnell, Farshad Farshidfar, Aalim M. Weljie, Karen A. Kopciuk, Elijah Dixon, Chad G. Ball, Francis R. Sutherland, Hans J. Vogel, Oliver F. Bathe

**Affiliations:** 1 Department of Oncology, University of Calgary, Calgary, AB T2N 4N2, Canada; yarrow.mcconnell@gmail.com (Y.J.M.); farshidf@ucalgary.ca (F.F.); karen.kopciuk@albertahealthservices.ca (K.A.K.); elijah.dixon@albertahealthservices.ca (E.D.); 2 Department of Surgery, University of Calgary, Calgary, AB T2N 4N2, Canada; ball.chad@gmail.com (C.G.B.); francis.sutherland@albertahealthservices.ca (F.R.S.); 3 Department of Biological Sciences, University of Calgary, Calgary, AB T2N 4N2, Canada; aalim@upenn.edu (A.M.W.); vogel@ucalgary.ca (H.J.V.); 4 Department of Pharmacology, University of Pennsylvania, Philadelphia, PA 19104, USA; 5 Department of Mathematics and Statistics, University of Calgary, Calgary, AB T2N 4N2, Canada

**Keywords:** biomarkers, metabolomics, pancreatic cancer, periampullary adenocarcinoma

## Abstract

Previous work demonstrated that serum metabolomics can distinguish pancreatic cancer from benign disease. However, in the clinic, non-pancreatic periampullary cancers are difficult to distinguish from pancreatic cancer. Therefore, to test the clinical utility of this technology, we determined whether *any* pancreatic and periampullary adenocarcinoma could be distinguished from benign masses and biliary strictures. Sera from 157 patients with malignant and benign pancreatic and periampullary lesions were analyzed using proton nuclear magnetic resonance (^1^H-NMR) spectroscopy and gas chromatography–mass spectrometry (GC-MS). Multivariate projection modeling using SIMCA-P+ software in training datasets (*n* = 80) was used to generate the best models to differentiate disease states. Models were validated in test datasets (*n* = 77). The final ^1^H-NMR spectroscopy and GC-MS metabolomic profiles consisted of 14 and 18 compounds, with AUROC values of 0.74 (SE 0.06) and 0.62 (SE 0.08), respectively. The combination of ^1^H-NMR spectroscopy and GC-MS metabolites did not substantially improve this performance (AUROC 0.66, SE 0.08). In patients with adenocarcinoma, glutamate levels were consistently higher, while glutamine and alanine levels were consistently lower. Pancreatic and periampullary adenocarcinomas can be distinguished from benign lesions. To further enhance the discriminatory power of metabolomics in this setting, it will be important to identify the metabolomic changes that characterize each of the subclasses of this heterogeneous group of cancers.

## 1. Introduction

Patients with masses and strictures of the pancreas or periampullary structures may present with jaundice or pain, or lesions can be found incidentally on diagnostic imaging. Once a lesion of the pancreas and periampullary region is identified, further diagnostic tests are required to determine whether the lesion is benign or malignant (most commonly adenocarcinoma). Malignant lesions warrant early surgical consideration. Benign lesions, such as pancreatitis, benign strictures, and serous cysts, are ideally treated non-operatively.

Despite an extensive diagnostic workup consisting of cross-sectional imaging, endoscopic retrograde cholangiopancreatography (ERCP) with brush biopsy, endoscopic ultrasound (EUS) with fine needle aspiration (FNA), and serum CA19-9, malignant lesions can be diagnosed definitively only 60%–90% of the time [1,2,3,4]. Importantly, there is no sure way to determine whether a lesion is benign. There are a number of related implications. The extensive diagnostic work (including invasive tests) may delay treatment for patients who are ultimately proven to have a pancreatic or periampullary cancer. In addition, a large proportion of patients undergo major surgery without a definitive diagnosis. This results in a finding of benign pathology in 7%–31% of pancreatic surgical resection specimens [5,6,7,8]. Given that pancreatic surgery is associated with substantial morbidity and a significant risk of perioperative mortality [9,10], a reduction in the need for such “diagnostic” resections would be beneficial.

Better non-invasive diagnostic tests that accurately discriminate malignant from benign pancreatic lesions are clearly needed. Metabolomics, like other ‘omics’ fields, explores the ability of multimarker panels to differentiate between disease states. Our previous work demonstrated the ability of proton nuclear magnetic resonance (^1^H-NMR) spectroscopy to differentiate serum samples from patients with pancreatic cancer versus benign pancreaticobiliary disease using 22 metabolites and achieving an internal AUROC of 0.83 [11]. The current study is an extension of that work. 

Firstly, we sought a more comprehensive evaluation of the metabolome by testing a larger group of samples using both ^1^H-NMR spectroscopy and gas chromatography–mass spectrometry (GC-MS). GC-MS has the potential to enhance the final metabolomic profile due to its greater sensitivity and ability to detect different metabolites than ^1^H-NMR spectroscopy [12]. ^1^H-NMR spectroscopy and GC-MS results were analyzed separately as well as in a combined fashion, to evaluate their relative strength and potential synergism. Secondly, we explored the application of metabolomics in a clinically relevant cohort of periampullary lesions, providing a more realistic analysis of the performance and limitations of a single metabolomic profile to distinguish benign from malignant disease. Previous studies have shown that the metabolomic profile of blood and urine can effectively distinguish benign disease from pancreatic cancer [11,13,14,15,16]. However, when located near the head of the pancreas, pancreatic cancer is mostly clinically indistinguishable from other periampullary adenocarcinomas. Therefore, it is unlikely that a test distinguishing benign disease from pancreatic cancer alone would be clinically useful unless it could also identify non-pancreatic periampullary cancers. To this end, using the two analytical modalities (^1^H-NMR spectroscopy and GC-MS), the minimal list of metabolites that consistently distinguished patients with malignant and benign pancreatic/periampullary lesions was identified in randomly allocated training sets, then validated in separate test sets.

## 2. Materials and Methods

### 2.1. Serum Samples

Venous blood samples were obtained from 157 patients who had a pancreatic or periampullary lesion on diagnostic imaging. All patients provided written consent consisting of permission to collect and bank blood and to collect linked demographic and clinical data for the purpose of supporting ethics-approved research projects. The procedures of the University of Calgary Hepatopancreaticobiliary/Gastrointestinal Tumor Bank, including consent, were approved by the Conjoint Health Research Ethics Board at the University of Calgary (Ethics ID E17213). The use of blood to identify metabolomic and proteomic biomarkers of pancreatic and periampullary tumors was also approved by the Conjoint Health Research Ethics Board at the University of Calgary (Ethics ID E20846). All patients had fasted for at least 8 hours at the time of sample collection.

For patients not undergoing surgical resection, samples were collected at a licensed laboratory collection facility. For patients undergoing surgical resection, samples were collected on the day of surgery, prior to any surgical manipulation. Serum samples were collected and stored as previously described [11].

### 2.2. Patient Data

Clinical data were collected prospectively as part of the serum banking process. Each patient was classified as having either a malignant or a benign pancreatic/periampullary lesion based on review of pathology, diagnostic imaging, and operative and clinic notes. Malignancies included adenocarcinomas of the pancreas, distal bile duct, ampulla of Vater and duodenum (all residing in the pancreatic and periampullary regions). In cases where finding the exact origin of an adenocarcinoma was not possible because the tumour was unresectable (*n* = 65), the lesion was classified according to the diagnosis favoured by the consulting surgeon based on the clinical course of the patient. For malignant lesions, stage classification was assigned according to the American Joint Committee on Cancer (AJCC) Cancer Staging Manual (7th Edition) [17].

### 2.3. Metabolomic Analysis

Serum samples for this study were analysed using ^1^H-NMR spectroscopy and GC-MS according to previously published protocols [11,18]. For ^1^H-NMR, a Bruker Avance 600 NMR spectrometer, operating at 600.22 MHz and equipped with a 5 mm TXI probe at 298 K was used. Spectra were acquired by using standard Bruker pulse sequence program (pr1d_noesy) in series of 1024 scans. Then 65,536 data points over the spectral width of 7211 Hz were then Fourier transformed using the Chenomx NMR Suite 6.1 processor software (Chenomx Inc., Edmonton, AB, Canada).

For GC-MS, each sample was randomly assigned to one of four sequential days for extraction, and then, with a separate randomization, assigned to one of four sequential days for derivatization and GC-MS analysis. Briefly, the modified Bligh and Dyer extraction and purification method for metabolite extraction [19]. For GC-MS analysis an Agilent 7890A chromatograph (Agilent Technologies Canada Inc., Mississauga, ON, Canada) equipped with an autosampler, was used. This was coupled with a Waters GCT Premier orthogonal acceleration/time-of-flight (oa-TOF) mass spectrometer (Waters Corp., Milford, MA, USA). An MS range of 50 to 800 *m/z* was used for scanning each sample in 31 min.

Metabolites from the ^1^H-NMR spectroscopy dataset were identified and quantified using the Human Metabolome Database (HMDB, version 2.5) [20] and Chenomx NMR Suite using the “Targeted Profiling” approach [21]. Metabolites and features from the GC-MS dataset were identified using Metabolite Detector software [22] (Version 2.06, Technische Universität Carolo-Wilhelmina zu Braunschweig, Braunschweig, Germany) and an in-house library based on the GOLM metabolite database [23]. All metabolite features, whether matched to an identification by Metabolite Detector or not, were included in the dataset for further analysis. Species matched to an entity in the GOLM database that does not yet have an associated chemical name were labelled with the word “Match”, their retention index (RI) value, and the list of *m*/*z* values for quantified ions (e.g., “Match: RI 1416.54, Ions 110 134 184 217 228”); and species not matched to any entity in the GOLM database were labelled with the word “Unmatched”, their RI value, and the list of *m*/*z* values for quantified ions (e.g., “Unmatched: RI 2475.33, Ions 73 375 376”).

### 2.4. Data Pre-Processing

All zero values were considered as missing values and all metabolites or features with >50% missing values were excluded from further analysis. The resulting ^1^H-NMR dataset contained 60 metabolites and the GC-MS dataset contained 123 metabolites/features for further analysis. Data pre-processing was conducted separately for the ^1^H-NMR spectroscopy and GC-MS datasets using STATA (version 12.0, StataCorp, College Station, TX, USA) and consisted of: median fold change normalization [24]; logarithmic transformation; centering; and unit variance scaling [25]. Median fold change normalization corrects for unwanted inter-sample differences in concentration and quality of preparation. Logarithmic transformation enhances the performance of the projection models that are based on the normality assumption for each metabolite, by making each metabolite’s distribution approach a normal distribution.

The resulting datasets had 22 metabolites in common (alanine, aspartate, citrate, glucose, glutamate, glutamine, glycerol, glycine, histidine, hypoxanthine, isoleucine, methionine, ornithine, phenylalanine, proline, pyroglutamate, serine, threonine, tryptophan, tyrosine, urea, and valine). For each of these metabolites, the ^1^H-NMR spectroscopy and GC-MS values was block normalized and included in a new combined dataset as previously reported [26]. To this was added the remaining 38 non-shared ^1^H-NMR metabolites and 101 non-shared GC-MS metabolites/features, giving a total of 161 metabolites/features in the combined dataset.

### 2.5. Multivariate Projection Modeling

Three random allocations of the 157 patient samples to training and test sets were conducted, in a 50:50 split, with separate stratifications within diagnosis class (malignant vs. benign) for serum sampling year (≤2008 vs. >2008), GC-MS extraction day (1 or 2 vs. 3 or 4), and GC-MS derivatization day (1 or 2 vs. 3 or 4).

SIMCA-P+ (Version 12.0, Umetrics, Umea, Sweden) software was used for all multivariate projection modeling. All modeling procedures were conducted separately for each of the three training sets, using the ^1^H-NMR spectroscopy, GC-MS, and combined datasets. Thus, a total of nine training models were generated (3 datasets x 3 trials). For each model, metabolites were pre-filtered using a *t*-test of distributions between malignant and benign lesions (*p*-value <0.3). Unsupervised principal component analysis (PCA) was then conducted to look for marked outliers and any latent structures within each model [27].

For each of the nine training sets, bidirectional orthogonal partial least squares (O2PLS) analysis was conducted using the following covariates: patient age, gender, lesion location, lesion type, surgical resection, cancer staging (where applicable), jaundice, diabetes mellitus, bowel cleansing, sampling year, and sampling location. For analysis of the GC-MS and combined datasets, extraction and derivatization days were added as covariates. Metabolites contributing more to the modeling of non-diagnostic covariates than to the modeling of the diagnostic class, were excluded iteratively until the diagnostic class was the covariate contributing most to the overall model. The resulting reduced list of metabolites was then submitted to orthogonal partial least squares-discriminant analysis (OPLS-DA) modeling. Using Variable Importance to Projection (VIP) values and coefficients, the list of metabolites was iteratively reduced to the absolute minimum required to maintain the strength of model parameters. For each training set, the resulting focused metabolite list was compiled and model parameters reported. The internal validity of the generated models was then tested by prediction of the diagnostic classification in the respective independent test sets, and area under the receiver operating curve (AUROC) values were calculated.

### 2.6. Metabolic Pathway Analysis

The focused list of metabolites from each trial was extracted, along with their respective regression coefficients and VIP values. These lists were combined for the three trials for each dataset. For metabolites found in the focused list for at least 2/3 of test data sets, average coefficient and VIP values were calculated. This yielded a focused list of metabolites for ^1^H-NMR spectroscopy, GC-MS, and combined datasets, respectively. A list of consistently contributing metabolites across all trials and datasets was compiled and submitted for topological metabolic pathway analysis using MetaboAnalyst software (version 2.0, Metabolomics Innovation Centre, Edmonton, AB, Canada) [28,29]. Where a metabolite was contributing to all three datasets, the average concentration/intensity data from the combined dataset was used. Otherwise, the concentration/intensity data from the ^1^H-NMR spectroscopy or GC-MS dataset was used, as appropriate.

## 3. Results

### 3.1. Demographic and Technical Factors

Lesions in the pancreas and periampullary region consisted of solid masses (*n* = 111), cystic lesions (*n* = 20), and biliary strictures (*n* = 19); seven were combinations of strictures and masses. Jaundice was present in 31 patients (19.7%), diabetes was present in 33 patients (21%). There were 35 benign lesions (22.2%). Of the malignant lesions, 84 (68.9%) were stage II and III cancers. All benign lesions were resected, 92 of the malignant lesions (75.4%) were resected. There were some differences in the benign and malignant groups. Average age was 57 ± 13 in the benign group, 66 ± 11 years in the malignant group (*p* < 0.0001). Jaundice was present in one patient with benign disease (2.8%), 30 patients with cancer (24.5%; *p* = 0.004). Finally, diabetes was present in six patients with benign lesions (17.1%) and 27 of patients with malignancy (22.1%; NS). For each of the three separate randomized allocations to the 50:50 split, the training group contained 80 patient samples, and the test group contained 77 patient samples. Clinical and technical factors appeared evenly distributed for each allocation, and training sets had similar characteristics as validation sets (Table 1).

### 3.2. Principal Component Analysis

**Figure 1:**
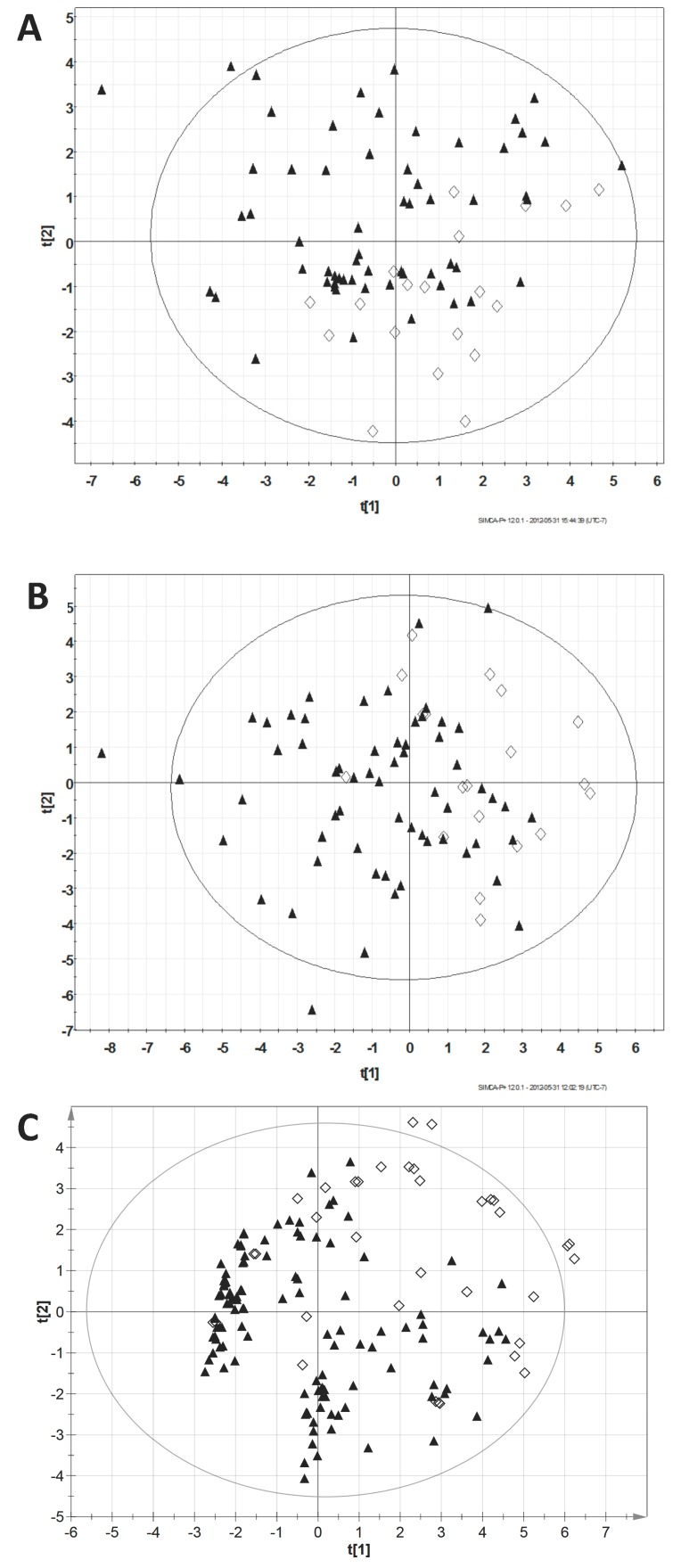
Principal components analysis (PCA) results. Scatter plots showing scores (t) in first two components of PCA models for one training dataset ((**A**) ^1^H-NMR; (**B**) GC-MS; (**C**) Combined). Results from other training sets were similar. Plots coded for patient diagnosis: malignant: ▲ vs. benign: ♢.

On PCA modeling, no marked latent structures were identified and no sample was a consistent outlier across allocation trials (Figure 1). The cumulative R^2^X for the NMR model was 0.31 (based on four components); for GC-MS it was 0.46 (based on nine components).

### 3.3. Orthogonal Multivariate Projection Modeling

**Figure 2:**
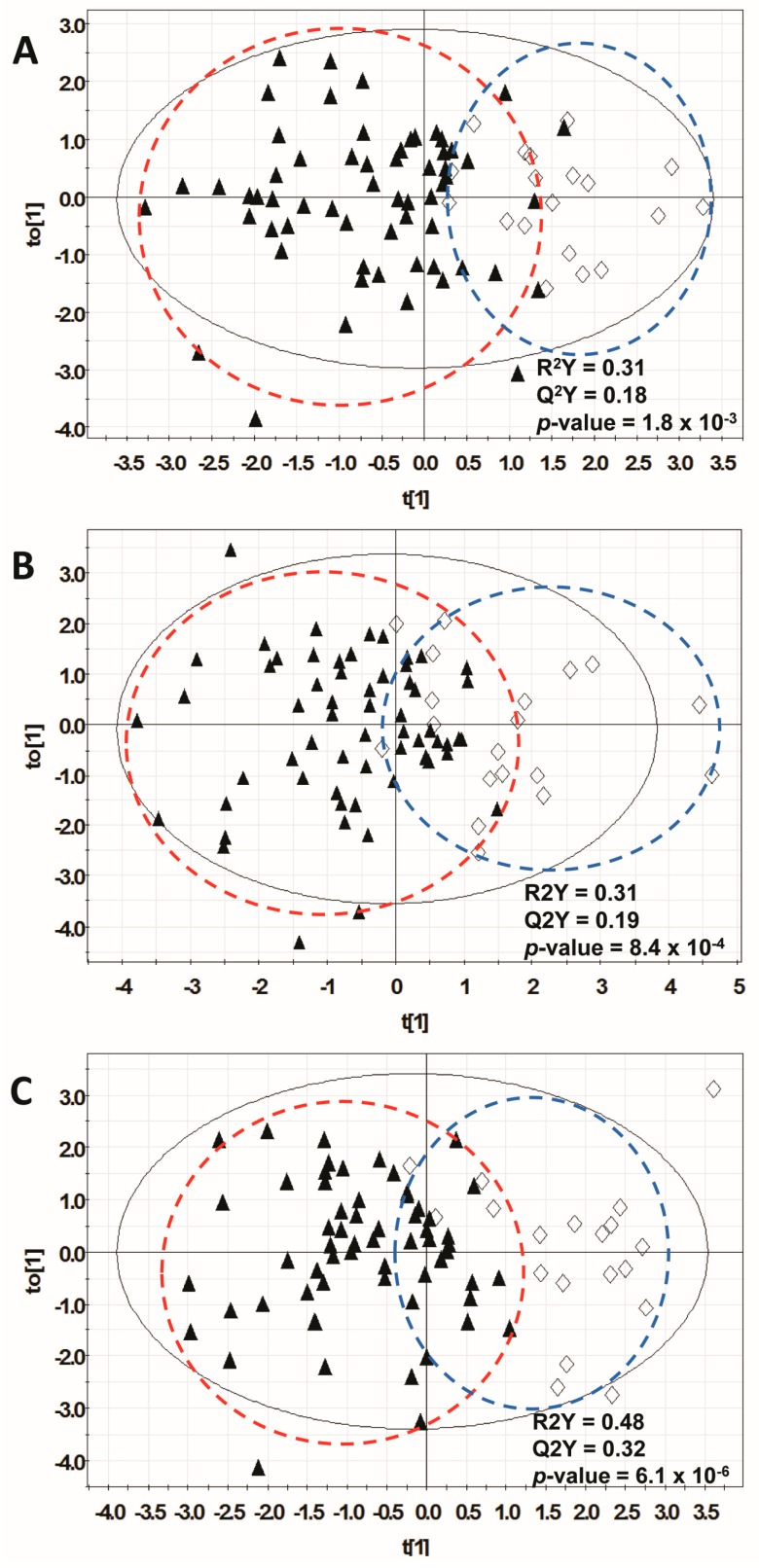
Orthogonal partial least squares discriminant analysis (OPLS-DA) results. Scatter plots showing scores (t) in first (t[1]) and orthogonal (to[1]) components of final OPLS-DA models for one training dataset (**A**) ^1^H-NMR; (**B**) GC-MS; (**C**) Combined). Results from other training sets were similar. Plots coded for patient diagnosis: malignant: ▲ vs. benign: ♢.

Table 2 summarizes the results of modeling for the ^1^H-NMR spectroscopy, GC-MS and combined datasets, and Figure 2 displays the respective scores plots. These results indicate the ability of metabolites from these three partitioned datasets to distinguish malignant versus benign lesions in training sets of 80 patient samples, with independent validation in test sets of 77 patient samples.

For the ^1^H-NMR spectroscopy dataset, the focused metabolite lists contained an average of 14 metabolites and the resulting models had the following average parameters: R^2^Y 0.308, Q^2^ 0.184, and CV-ANOVA p value 1.8 × 10^−3^. On independent validation in the test sets, the average AUROC was 0.74 (SE = 0.06). When the same training sets were tested in the GC-MS dataset, the average focused metabolite list contained 18 compounds. Average model parameters were R^2^Y 0.312, Q^2^ 0.188, and CV-ANOVA p value 8.4 × 10^−4^. On independent validation in the respective test sets, the mean AUROC was 0.62 (SE = 0.08). For both H-NMR and GC-MS, there is less variability in the benign samples than in the malignant samples. The malignant group is comprised entirely of adenocarcinomas, but the tissue of origin is heterogeneous, which may partly contribute to the larger degree of variability. In addition, the benign group is smaller, so there is limited variability expected in these circumstances. We do not believe that we have introduced any systematic bias, as we randomized the sample analysis.

For the combined dataset, focused metabolite lists contained, on average, 20 metabolites and the resulting models had, on average, R^2^Y 0.478, Q^2^ 0.324, and CV-ANOVA p value 6.14 × 10^−6^. On validation in the respective test sets, the average AUROC was 0.66 (SE = 0.08). When the model containing only the eight metabolites in common was tested, the AUROC was 0.72 ± 0.05.

Eight metabolites were found to consistently contribute to the malignant/benign profile across all three datasets (Table 3): higher levels of glutamate, myo-inositol, phenylalanine, and urea were consistently correlated with malignancy; while higher levels of glutamine, ornithine, proline, and threonine were consistently correlated with benign disease. An additional 22 metabolites were found less consistently across the datasets. These metabolites were identified in the modeling for at least two trials in at least one dataset, or in different trials in different datasets. Of these, nine metabolites were associated with malignancy and 13 metabolites were associated with benign disease (Table 3).

Whisker plots of the raw data for all 30 consistently contributing metabolites are included as Supplementary Information. As in our previous clinical studies, the differences in individual metabolites between disease states are small. However, it is the pattern of multiple co-related metabolites that differ, as indicated in the above descriptions of OPLS-DA models. Thus, no single metabolomics feature is particularly informative, but the meta-biomarker comprised of co-relationships is reflective of disease state.

### 3.4. Metabolic Pathway Analysis

The predominant differences between malignant and benign patient samples appeared to occur within amino acid and carbohydrate metabolic pathways (Table 4). The arginine/proline pathway had the largest impact factor (0.456, *p* = 0.000085) with consistently higher levels of arginine, creatine, glutamine, ornithine, and proline seen in the benign samples and consistently higher levels of glutamate and urea in malignant samples. The alanine/aspartate/glutamate pathway had the next largest impact factor (0.441, *p* = 0.00026), reflecting the consistently higher levels of alanine and glutamine in benign samples versus glutamate and succinate in malignant samples. Galactose levels were higher in malignant samples and the galactose metabolism pathway had the third largest impact factor (0.224, *p* = 0.000086). The list of all statistically impacted pathways is included in Table 4.

## 4. Discussion

Clinically, to make timely treatment decisions, it would be beneficial to have a noninvasive test that distinguishes a benign from a malignant pancreatic and periampullary mass or stricture. A number of investigators have demonstrated that the serum metabolomic profile can discriminate pancreatic cancer from benign pancreatic lesions [11,13,14,15]. However, in the clinic, it is not always possible to distinguish pancreatic cancer from other periampullary adenocarcinomas. Therefore, our goal was to identify the metabolomic features that separated all pancreatic and periampullary adenocarcinomas from benign masses and strictures using a two-class model. This was considered feasible because cancers have some common features to their disordered metabolism, such as the Warburg effect.

We determined that focused metabolomic profiles containing as few as 14–18 metabolites do discriminate between serum samples from patients with malignant versus benign pancreatic/periampullary lesions. In the training set, these focused metabolomic profiles produced OPLS-DA models with R^2^ values of 0.30–0.48, indicating that 30%–48% of the observed variance in metabolite levels was attributable to the diagnostic classification. These values are in the range expected for clinical specimens [30], are in keeping with the clustering of samples by diagnostic category seen in the first and second components of unsupervised PCA, and were sufficient to statistically discriminate between diagnostic classes as indicated by the CV-ANOVA p-values. In separate validation sets, the AUROC values of metabolomics models had a level of performance similar to that of the serum tumor marker CA 19-9, suggesting that the metabolomic profile may have some value [1]. However, the test performance is insufficient to have a direct impact on clinical decision-making in its present form.

Our metabolomic models did not perform as well as in other studies, including our own. This is because our comparator groups consisted of disease processes that were more heterogenous than in other series, which typically consisted of pancreatic adenocarcinoma versus pancreatitis and/or or normal controls [11,13,14,15,31,32,33]. This was by design. Clinically, pancreatic and non-pancreatic adenocarcinomas are frequently indistinguishable. (It is only after resection that they can be definitively classified.) Therefore, if one were to apply a metabolomic profile for pancreatic cancer (as described by previous investigators) to a clinical population, it would underperform because of the inherent heterogeneity of the metabolomic features of similar lesions. We wanted to see if there was a simple “adenocarcinoma profile” that might be more applicable. The inferior performance of our model was not completely surprising, given that pancreatic cancer is associated with diabetes and non-pancreatic periampullary adenocarcinomas are not. Our experimental design provides a more realistic estimate of the performance of a test based on a single two-class metabolomic model. To enhance the performance of a metabolomics-based blood test for identification of individuals with malignancy, it will be important to define the metabolomic alterations associated with pancreatic and non-pancreatic adenocarcinomas separately.

The results of previous studies may also be overly optimistic because external validation sets were not always reported. In most studies, excellent AUROCS have been reported when metabolomic models were internally validated [11,15], exaggerating the performance of the metabolomic models. When external cohorts are used for validation, AUROCs on the validation sets are typically lower than in internal validation cohorts. For example, Kobayashi et al. compared metabolomic profiles of sera from pancreatic cancer and chronic pancreatitis analyzed by GC-MS [14]. They described an AUROC of 0.93 in a training set, and the AUROC in the validation set was only 0.76, which was no better than CA19-9 (AUROC 0.82) and CEA (AUROC 0.80). This illustrates the critical need for validation sets wherever possible in this field.

Unexpectedly, combining two complimentary analytical modalities in an attempt to gain a more comprehensive interrogation of the metabolome did not markedly improve test performance. We had hypothesized that creating a combined ^1^H-NMR spectroscopy/GC-MS dataset could harness the relative strengths of each platform, providing stronger predictions. However, the combined dataset models performed only slightly better than the GC-MS models and not as well as the ^1^H-NMR models. The combined metabolomic model derived from ^1^H-NMR spectroscopy and GC-MS was limited by the stability of the latter. ^1^H-NMR spectroscopy models generally performed better than the GC-MS models, with a smaller standard error for the AUROC values. In addition, the average standard error for the metabolite coefficients was 62% higher for GC-MS compared to ^1^H-NMR spectroscopy, indicating more variability in the regression modeling of metabolites in the GC-MS model. Finally, the consistency of metabolites identified across allocation sets was higher for ^1^H-NMR spectroscopy than for GC-MS. For ^1^H-NMR spectroscopy, 58% of metabolites important to the final focused list were identified in two or more of the allocation sets; only 36% of metabolites in the final list were identified in two or more of the allocation sets in GC-MS (*p* = 0.04).

It is possible that alternative approaches will be required to merge data from two analytical platforms. In the present study, a simple averaging technique was used to combine metabolites detected by both platforms. Other approaches to combining data are being developed and used, but no standard approach has yet been established [34,35]. Further work in this field may result in a method that effectively capitalizes on the relative strengths of multiplatform detection, to produce an even stronger diagnostic model.

Compared to our previous study using ^1^H-NMR spectroscopy to distinguish pancreatic cancer from benign tumors, some of the same changes in metabolites were observed in the present study. Specifically, in both studies, malignancy was associated with elevated glutamate, phenylalanine, and mannose; creatine, glutamine, threonine, and lysine were higher in the benign condition. These metabolites did not vary significantly by sex or any other factor other than disease state. The fact that these changes were seen in independent studies, despite the heterogeneity of the cancers included in the present study is encouraging.

The metabolomic profiles identified offer insights into the metabolomic pathways altered in patients with a pancreatic and periampullary malignancy. The results clearly indicate a tipping of the balance of amino acid metabolism towards higher glutamate levels in malignant samples and higher glutamine and alanine levels in benign disease. These observations are consistent with earlier findings published by our group, which found elevated levels of glutamate in the serum of pancreatic cancer patients when compared to that of patients with benign pancreatic or biliary disease [11]. It is also consistent with findings in many cancer model systems that show a switch to glutamine consumption, and increased glutamate and succinate production, in patients with rapidly proliferating cancer cells, as part of the “Warburg effect” [36,37].

Arginine and ornithine are part of the urea cycle and feed the production of putrescine, the rate-limiting step in protein synthesis. The conversion of arginine to ornithine, by the enzyme arginase, has been suggested as a major regulator of cell growth [36]. It is therefore interesting that arginine and ornithine levels were lower in the serum of patients with pancreatic cancer compared to benign pancreatic lesions. The level of urea, a side product of arginine-to-ornithine conversion, was slightly higher in patients with pancreatic cancer. Together, these findings are consistent with altered arginase activity in patients with pancreatic and periampullary adenocarcinomas. In pancreatic cancer samples tested by the International Cancer Genome Consortium [38], only 1% contained ARG1 mutations, and no ARG2 mutations were seen. Therefore, the mechanism for this observation must be further elucidated.

The correlation of serum galactose with pancreatic cancer is seen in the GC-MS dataset only, as NMR did not detect galactose in these conditions. A similar relationship was recently observed with colorectal cancer patients [18], but further investigation is needed before any putative mechanism can be proposed.

## 5. Conclusions

In conclusion, it is possible to distinguish benign and malignant pancreatic/periampullary masses and biliary strictures using ^1^H-NMR and GC-MS based on a two-class metabolomic model. We speculate that even better test performance could be expected if the metabolomic features of various pancreatic and periampullary cancers were defined, discriminating disease state on a multi-class model. Current methods of combining the employed analytical modalities do not enhance diagnostic power. However, if a more comprehensive analysis of the metabolome were done using quantitative methods (as opposed to semi-quantitative methods) or more sensitive techniques, perhaps targeting metabolites identified in discovery efforts such as this, one may derive a more robust diagnostic test. Using an analytic modality with broader coverage and improved separation, such as liquid chromatography-mass spectrometry, may also augment test performance.

## Figures and Tables

**Table 1:** Clinical and technical variables for each allocation of training and test sets.

		Allocation A			Allocation B			Allocation C		
		Training *n* = 80	Test *n* = 77	*p* *	Training *n* = 80	Test *n* = 77	*p*	Training *n* = 80	Test *n* = 77	*p* *
Age	<60 yrs	24	24	0.87	27	21	0.38	22	26	0.40
	≥60 yrs	56	53		53	56		58	51	
Gender	Male	45	37	0.31	46	36	0.18	40	42	0.57
	Female	35	40		34	41		40	35	
Lesion Location	Head/Uncinate	52	58	0.31	54	56	0.59	55	55	0.46
	Body/Tail	20	15		19	16		20	15	
Lesion Type	Mass	58	53	0.37	59	52	0.57	54	57	0.12
	Stricture	8	11		7	12		13	6	
	Cyst	9	11		11	9		12	8	
*Diagnosis*	*Malignant*	*61*	*61*	*0.61*	*61*	*61*	*0.61*	*61*	*61*	*0.61*
	*Benign*	*19*	*16*		*19*	*16*		*19*	*16*	
Stage (for Malignant Lesions Only)	I	11	8	0.39	5	14	0.24	12	7	0.25
	II	27	28		30	25		27	28	
	III	16	13		18	11		14	15	
	IV	7	12		8	11		8	11	
Surgically Resected	Yes	48	44	0.72	43	49	0.21	49	43	0.49
	No	32	33		37	28		31	34	
Jaundice	Yes	13	18	0.26	14	17	0.47	15	16	0.75
	No	67	59		66	60		65	61	
Diabetes Mellitus	Yes	20	13	0.21	17	16	0.94	18	15	0.64
	No	60	64		63	61		62	62	
Bowel Cleansing	Yes	43	43	1.0	42	44	0.30	47	39	0.46
	No	25	25		29	21		24	26	
*Sampling Year*	*2006-8*	*45*	*44*	*0.91*	*45*	*44*	*0.91*	*45*	*44*	*0.91*
	*2009-10*	*35*	*33*		*35*	*33*		*35*	*33*	
Sampling Location	Laboratory	12	17	0.25	17	12	0.36	11	18	0.12
	OR	68	60		63	65		69	59	
*GC-MS Extraction*	*Day 1/2*	*42*	*38*	*0.69*	*42*	*38*	*0.69*	*42*	*38*	*0.69*
	*Day 3/4*	*38*	*39*		*38*	*39*		*38*	*39*	
*GC-MS Derivatization*	*Day 1/2*	*40*	*41*	*0.68*	*40*	*41*	*0.68*	*40*	*41*	*0.68*
	*Day 3/4*	*40*	*36*		*40*	*36*		*40*	*36*	

* *p* values are for Mann-U-Witney testing between subgroups. *Italicized* variables were used as stratification factors in the randomized allocation process.

**Table 2:** Results of orthogonal partial least squares discriminant analysis (OPLS-DA).

Dataset	Mean of Training Sets (*n* = 80 each)				Mean of Test Sets (*n* = 77 each)	
	X	R^2^	Q^2^	*p*	AUROC	SE
^1^H-NMR	14	0.308	0.184	1.80 × 10^−3^	0.74	0.06
GC-MS	18	0.312	0.188	8.40 × 10^−4^	0.62	0.08
Combined *	20	0.478	0.324	6.14 × 10^−6^	0.66	0.08

* The combined dataset includes metabolite features from both ^1^H-NMR and GC-MS data, with averaged values for metabolites detected by both platforms. X: Mean number of unique metabolites/features in the focused metabolite lists across three randomized allocations of training/test set assignment; R^2^: goodness of fit; Q^2^: predictive ability of model (7-fold internal cross validation); *p*: *p*-value for CVANOVA testing; AUROC: area under the receiver operating curve; SE: standard error.

**Table 3:** Summary list of metabolite features included in final focused models.

	Metabolite	Datasets	Mean Coeff	Mean SE (Coeff)	Mean VIP	Mean SE (VIP)	*p*-Value in NMR	*p*-Value in GC-MS
**Higher in Malignant**	Galactose	G, C	0.121	0.069	1.123	0.683	-	0.001
	Unmatched RI:1007.82 QI: 67, 82, 83	G, C	0.120	0.074	1.337	0.708	-	0.11
	Isopropanol	N, C	0.114	0.042	1.001	0.382	0.01	-
	Phenylalanine	N, G, C	0.109	0.057	1.052	0.621	0.004	0.15
	Glutamate	N, G, C	0.105	0.064	1.127	0.616	0.01	0.01
	Mannose	N, C	0.102	0.069	1.220	0.410	0.01	-
	Trimethylamine-N-oxide	N	0.092	0.061	0.867	0.503	0.08	-
	Arabitol	G, C	0.090	0.047	0.967	0.409	-	0.16
	Threitol	G, C	0.088	0.080	0.889	0.816	-	0.14
	Succinate	N, C	0.086	0.115	0.743	0.777	-	-
	Urea	N, G, C	0.074	0.058	0.965	0.604	0.08	0.19
	Myo-Inositol	N, G, C	0.070	0.061	0.991	0.582	0.04	0.16
	Trehalose-alpha	G, C	0.059	0.053	0.624	0.572	-	0.21
**Higher in Benign**	Match RI:2018.25 QI: 191, 217, 305, 318, 507	G, C	−0.029	0.055	0.568	0.680	-	0.79
	Tridecanol	G	−0.060	0.051	0.738	0.613	-	0.28
	Azelaic acid	G	−0.061	0.038	0.814	0.526	-	0.04
	Unmatched RI:2475.33 QI: 73, 375, 376	G, C	−0.066	0.048	0.791	0.475	-	0.01
	Pyroglutamate	N	−0.068	0.036	0.696	0.306	0.18	-
	Isoleucine	G	−0.069	0.091	0.778	1.069	-	0.05
	Tyrosine	N, G	−0.074	0.058	0.862	0.669	0.21	0.08
	Arginine	N, C	−0.080	0.055	0.721	0.500	0.38	-
	Unmatched RI:1913.88 QI: 156, 174, 317	G, C	−0.090	0.067	1.092	0.863	-	0.01
	Proline	N, G, C	−0.096	0.063	1.009	0.547	0.03	0.10
	Alanine	N, C	−0.098	0.041	0.853	0.311	0.01	-
	Ornithine	N, G, C	−0.104	0.068	0.997	0.687	0.06	0.07
	Creatine	N, C	−0.107	0.041	0.952	0.267	0.06	-
	Glutamine	N, G, C	−0.115	0.072	1.107	0.686	0.0002	0.0001
	Lysine	N, C	−0.117	0.037	1.289	0.345	0.01	-
	Threonine	N, G, C	−0.137	0.065	1.360	0.538	0.04	0.001
	Unmatched RI:1971.99 QI: 185, 247, 275	G, C	–0.138	0.069	1.346	0.640	-	0.03

N: ^1^H-nuclear magnetic resonance spectroscopy, G: gas chromatography mass spectrometry, C: combined dataset, Coeff: regression coefficient for given X variable (metabolite) in the modeled Y variable (malignant versus benign), positive values associated with malignancy and negative values associated with benign disease; SE: standard error; RI: retention index, QI: quantification ions; VIP: variable importance to projection expresses overall contribution to the model. Metabolite features in *italics* were found in the focused lists for all three datasets.

**Table 4:** Topological metabolic pathway analysis.

Metabolic Pathway	Total Compounds in Pathway	Hits in Current Dataset	*p*	Impact Factor
Arginine and proline metabolism	77	7	8.49 × 10^−5^	0.456
Alanine, aspartate, and glutamate metabolism	24	4	2.60 × 10^−4^	0.441
Galactose metabolism	41	3	8.63 × 10^−5^	0.224
Lysine degradation	47	1	4.09 × 10^−3^	0.147
D-Glutamine and D-glutamate metabolism	11	2	1.37 × 10^−3^	0.139
Inositol phosphate metabolism	39	1	3.00 × 10^−2^	0.137
Phenylalanine metabolism	45	3	6.60 × 10^−3^	0.119
Aminoacyl-tRNA biosynthesis	75	10	8.90 × 10^−7^	0.113
Lysine biosynthesis	32	1	4.09 × 10^−3^	0.100
Glycine, serine and threonine metabolism	48	2	7.07 × 10^−4^	0.097
Tyrosine metabolism	76	2	2.77 × 10^−2^	0.047
Taurine and hypotaurine metabolism	20	1	8.27 × 10^−3^	0.032
Fructose and mannose metabolism	48	1	1.56 × 10^−3^	0.029
Butanoate metabolism	40	2	6.28 × 10^−3^	0.018
Valine, leucine, and isoleucine biosynthesis	27	2	9.74 × 10^−4^	0.013
Glutathione metabolism	38	3	3.35 × 10^−3^	0.013
Phenylalanine, tyrosine, and tryptophan biosynthesis	27	2	1.05 × 10^−2^	0.008
Purine metabolism	92	2	5.70 × 10^−4^	0.008

Produced using MetaboAnalyst software. For each pathway, the total number of known metabolites, along with the number of those found in the current dataset (“hits”) are reported. The *p* value is reported for the statistical comparison of metabolite feature levels between malignant and benign samples. The impact factor expresses the degree of centrality of the identified changes to the pathway functioning overall.

## Supplementary Materials

The following are available online at www.mdpi.com/2218-1989/7/1/3/s1, Figure S1: Principal Component Analysis (PCA) Scores Scatter plots for three training and validation set allocations of ^1^H-NMR, GC-MS and combined datasets; Figure S2: Receiver Operating Characteristic (ROC) curve for detection of benign and malignant periampullary lesions; Figure S3: Whisker plots for consistently contributing metabolites from NMR and GC-MS; Figure S4: Metaboanalyst 2.0 pathway analysis bubble plot; Table S1: Performance measurements in triplicate analysis of ^1^H-NMR dataset, 50/50 training/test dataset (*n* = 80/77); Table S2: Performance measurements in triplicate analysis of GC-MS dataset, 50/50 training/test dataset (*n* = 80/77); Table S3: Performance measurements in triplicate analysis of Combined dataset, 50/50 training/test dataset (*n* = 80/77).
