# Supplementary material for: Distinguishing Benign from Malignant Pancreatic and Periampullary Lesions Using Combined Use of 1H-NMR Spectroscopy and Gas Chromatography–Mass Spectrometry
Source: Metabolites. 2017 Jan 13;7(1):3. doi: 10.3390/metabo7010003 (PMC5372206; doi:10.3390/metabo7010003)
Supplement: Supplementary file 1 [file metabolites-07-00003-s001.pdf]

Supplementary Materials: Distinguishing Benign from Malignant Pancreatic and Periampullary Lesions Using Combined Use of ^{1}H-NMR Spectroscopy and Gas Chromatography–Mass Spectrometry

Yarrow J. McConnell, Farshad Farshidfar, Aalim M. Weljie, Karen A. Kopciuk, Elijah Dixon, Chad G. Ball, Francis R. Sutherland, Hans J. Vogel and Oliver F. Bathe

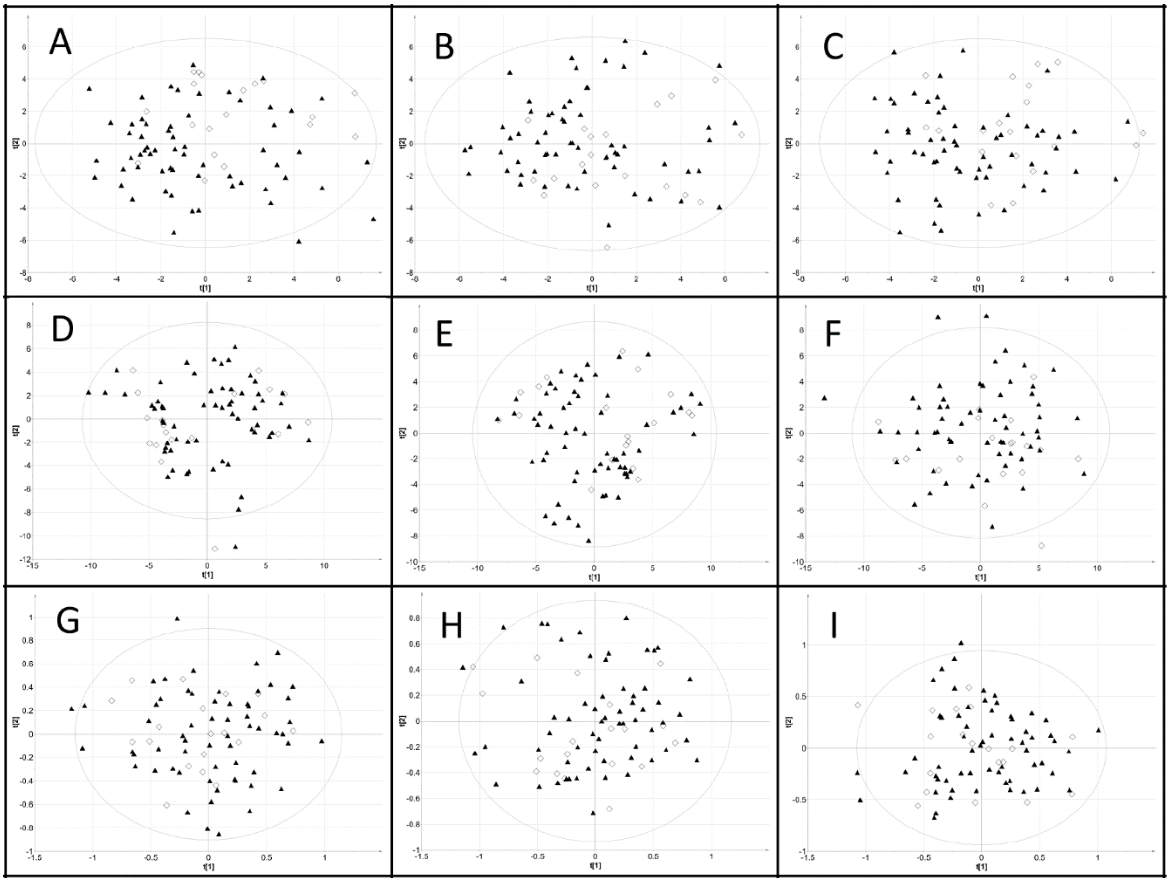

Figure S1. Principal Component Analysis (PCA) Scores Scatter plots for three training and validation set allocations of ^{1}H-NMR, GC-MS and combined datasets. All variables are included in the analyses. A, B, and C: three case allocations for ^{1}H-NMR data; D, E and F: three case allocations for GC-MS data; G, H and I: three case allocations for combined ^{1}H-NMR and GC-MS data. Sample sizes were 80 per allocation. Number of variables in each PCA (k) was 83 for ^{1}H-NMR spectroscopy; 146 for GC-MS; and 183 for combined. A= number of components.

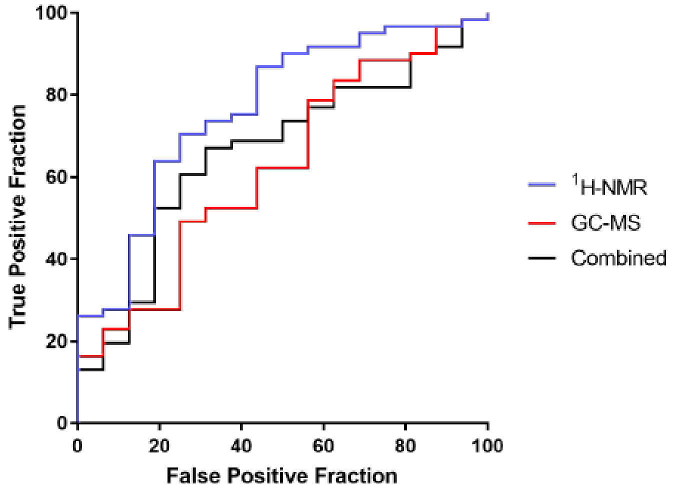

Figure S2. Receiver Operating Characteristic (ROC) curve for detection of benign and malignant periampullary lesions. ^{1}H-NMR: blue, GC-MS: red, Combined model: black.

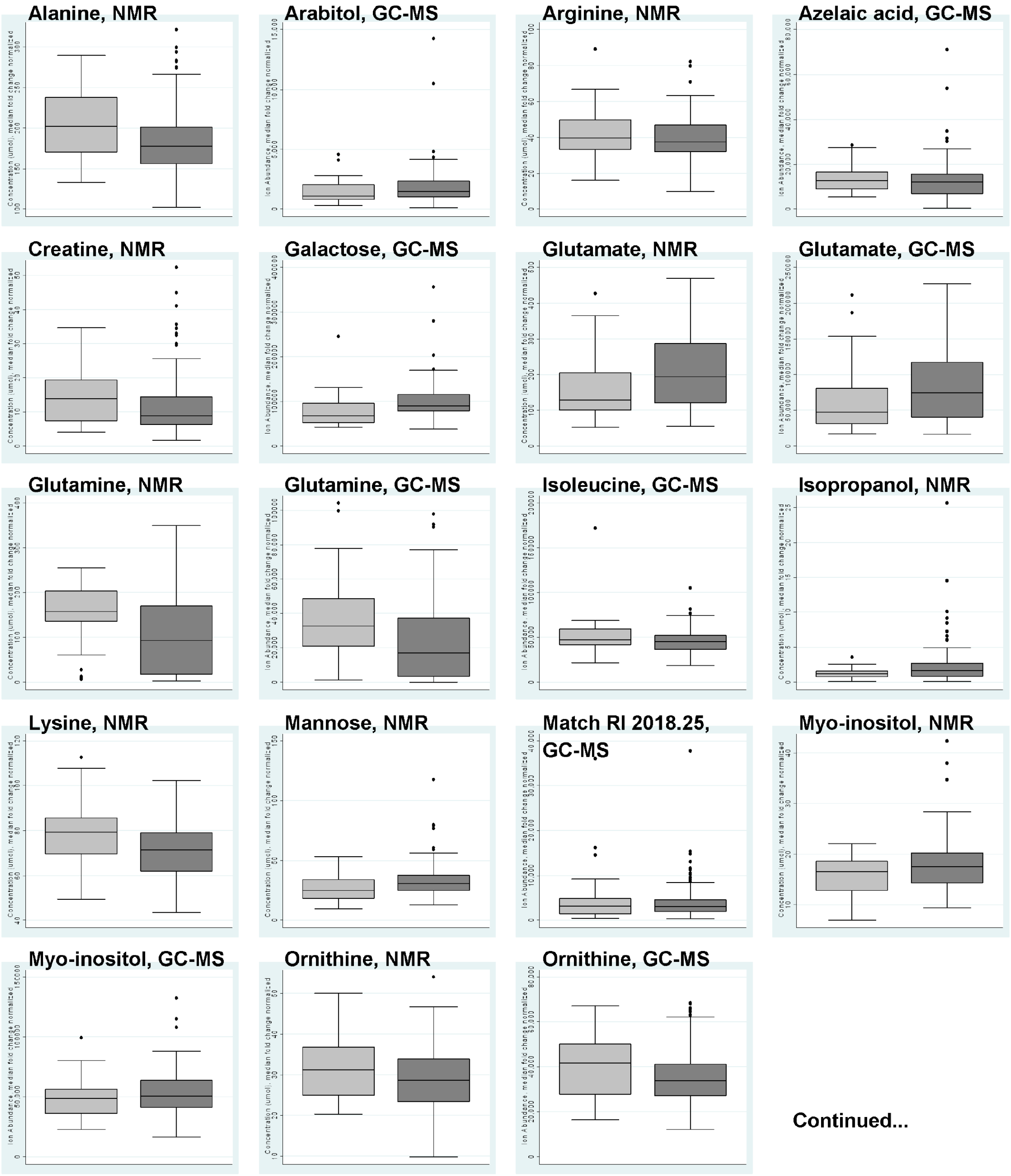

Figure S3. *Cont.*

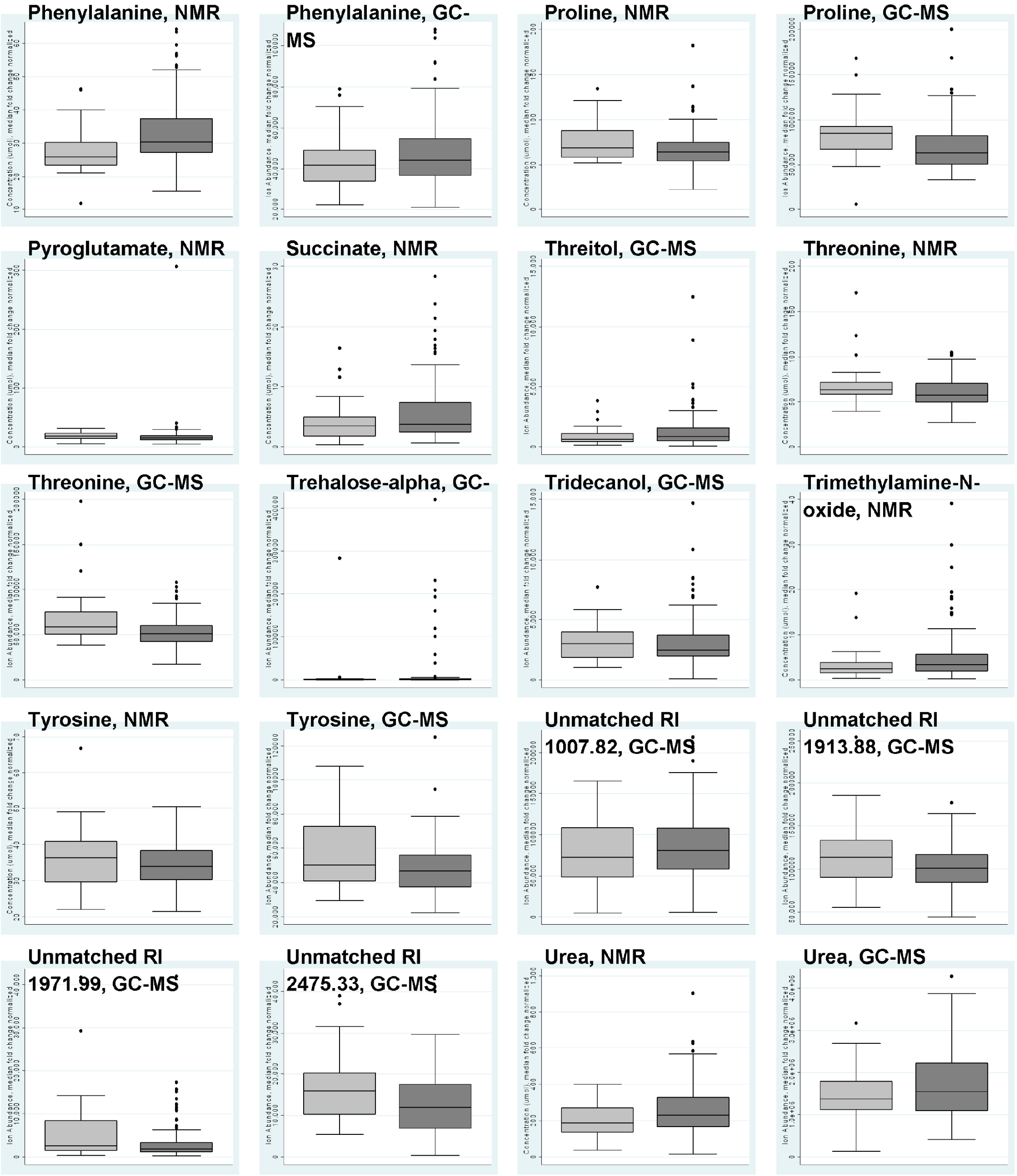

Figure S3. Whisker plots for consistently contributing metabolites from NMR and GC-MS. Whisker box limits are 1st and 3rd quartiles, whisker bar limits are upper and lower adjacent values. Middle line is median. Y-axis displays normalized concentration/ion abundance data: NMR = μmol, GC-MS = ion abundance. Benign disease: light gray, malignant disease: dark gray.

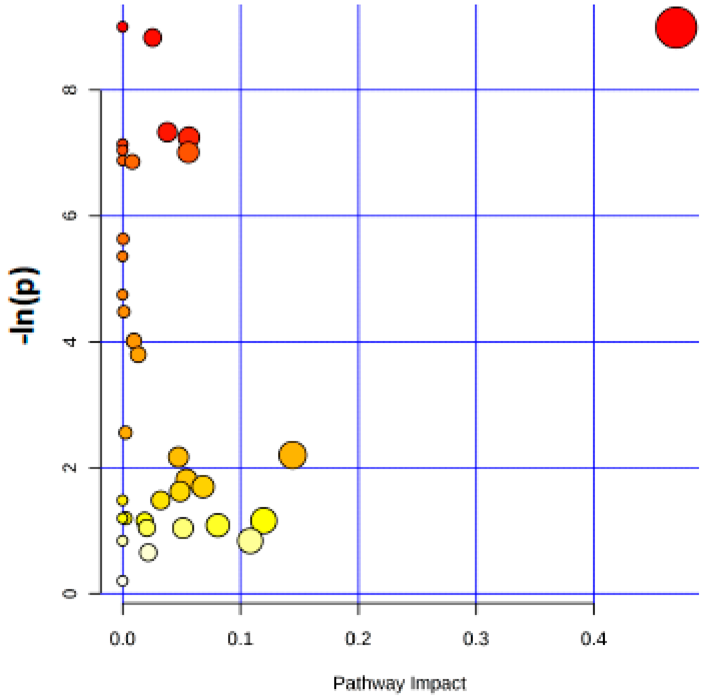

Figure S4. Metaboanalyst 2.0 pathway analysis bubble plot. The plot depicts each pathway's significance (expressed as "–ln(p-value)" on Y-axis) versus its degree of involvement in the biological process (as identified by "impact" on X-axis).

Table S1. Performance measurements in triplicate analysis of ^{1}H-NMR dataset, 50/50 training/test dataset (n = 80/77).

	**OPLS-DA: Filtered**							**OPLS-DA: Minimal**[c]								
	**Training**					**Test**		**Training**							**Test**	
**Trial**	**X**[a]	**R2Y**	**Q2**	***p***[b]	**Core CV**[c]	**AUROC**	**SE**	**X**[a]	**R2Y**	**Q2**	***p***[b]	**AUROC(i)**	**Cancer**	**Non-cancer**	**AUROC**	**SE**
**A**	26	0.334	0.174	6.4×10^{-4}	0.487	0.71	0.07	13	0.251	0.122	6.6×10^{-3}		Isopropanol<br>Mannose<br>Phenylalanine<br>Succinate<br>TrimethylamineNoxide<br>TwoHydroxyisobutyrat	Alanine<br>Betaine<br>Glutamine<br>Histidine<br>Lysine<br>Proline<br>Threonine	0.79	0.06
**B**	28	0.378	0.259	9.8×10^{-6}	0.484	0.73	0.07	14	0.308	0.224	6.1×10^{-5}		Mannose<br>MyoInositol<br>Phenylalanine<br>Urea	Creatine<br>Ethanol<br>Glutamine<br>Histidine<br>Lysine<br>Ornithine<br>Proline<br>Pyroglutamate<br>Threonine<br>Xanthine	0.71	0.05
**C**	24	0.282	0.140	3.3×10^{-3}	0.491	0.80	0.05	12	0.230	0.128	5.2×10^{-3}	0.756	*Choline*<br>*Isopropanol*<br>*Mannose*<br>*Succinate*<br>*TrimethylamineN-oxide*	Alanine<br>Creatine<br>Inosine<br>Lysine<br>Ornithine<br>Pyroglutamate<br>Threonine	0.75	0.06
Average	*26*	*0.331*	*0.191*	1.3×10^{-3}		*0.75*	*0.06*	*13*	*0.263*	*0.158*	3.8×10^{-3}				*0.75*	*0.06*

[a] Number of metabolites/features in the filtered (*t*-test *p*-value <0.3) training set

[b] *p*-value for CV-ANOVA testing

[c] Minimal list of metabolites determined by multistep iterative process: selection of metabolites contributing most to class difference by multivariate O2PLS analysis controlling for technical and clinical covariates; then iterative OPLS-DA modeling with sequential exclusion of metabolites based on their contribution to test model parameters- to maximize AUROC.

Table S2. Performance measurements in triplicate analysis of GC-MS dataset, 50/50 training/test dataset (n = 80/77).

	OPLS-DA: Filtered							OPLS-DA: Minimal[c]								
	Training					Test		Training							Test	
**Trial**	**X[a]**	**R2Y**	**Q2**	***p*[b]**	**Core CV[c]**	**AUROC**	**SE**	**X[a]**	**R2Y**	**Q2**	***p*[b]**	**AUROC(i)**	**Cancer**	**Non-cancer**	**AUROC**	**SE**
A	33	0.440	0.239	4.8×10^{-5}	0.736	0.69	0.07	17	0.336	0.165	1.3×10^{-3}	0.770	Arabitol5TMS<br>Galactose1MEOX<br>MatchRI170922<br>Threitol4TMS<br>Trehalosealpha<br>UnmatchedRI11763	Isoleucine2TMS<br>MatchRI129451<br>Ornithine4TMS<br>Proline2TMS<br>Sorbitol6TMS<br>Threonine3TMS<br>Tridecan1oln1TMS<br>UnmatchedRI16313<br>UnmatchedRI19719<br>UnmatchedRI24753<br>UnmatchedRI28215	0.69	0.07
B	43	0.378	0.258	1.3×10^{-5}	0.734	0.52	0.08	21	0.327	0.238	2.9×10^{-5}	0.832	Glutamicacid3TMS<br>Inositolmyo6TMS<br>MatchRI201825<br>Phenylalanine2TMS<br>UnmatchedRI10078<br>UnmatchedRI11250<br>UnmatchedRI11396<br>UnmatchedRI18425<br>Urea2TMS	AzelaicAcid2TMS<br>GlutamineDL3TMS<br>Glycerol3TMS<br>Octadecadienoic<br>Ornithine4TMS<br>Tridecan1oln1TMS<br>UnmatchedRI11646<br>UnmatchedRI13319<br>UnmatchedRI19138<br>UnmatchedRI19719<br>UnmatchedRI22199<br>UnmatchedRI24753	0.54	0.07
C	33	0.348	0.199	2.6×10^{-4}	0.784	0.70	0.07	17	0.273	0.162	1.2×10^{-3}	*0.755*	*Butanoicacid2am*<br>*Galactose1MEOX*<br>*Inositolmyo6TMS*<br>*Ribitol5TMS*<br>*Threitol4TMS*<br>*UnmatchedRI10078*<br>*Urea2TMS*<br>*UnmatchedRI22127*	AzelaicAcid2TMS<br>GlutamineDL3TMS<br>Isoleucine2TMS<br>MatchRI201825<br>Ornithine4TMS<br>Tyrosine3TMS<br>UnmatchedRI19138<br>UnmatchedRI19736<br>UnmatchedRI24753	0.67	0.08
Average	*36*	*0.389*	*0.232*	*1.1×10^{-4}*	*0.751*	*0.64*	*0.07*	*18*	*0.312*	*0.188*	*8.4×10^{-4}*	*0.786*			*0.67*	*0.07*

[a] Number of metabolites/features in the filtered (*t*-test *p*-value <0.3) training set
[b] *p*-value for CV-ANOVA testing
[c] Minimal list of metabolites determined by multistep iterative process: selection of metabolites contributing most to class difference by multivariate O2PLS analysis controlling for technical and clinical covariates; then iterative OPLS-DA modeling removing metabolites based on their contribution to predictive modeling in test set (YpredPS and T2PS)

Table S3. Performance measurements in triplicate analysis of Combined dataset, 50/50 training/test dataset (n = 80/77).

	OPLS-DA: Filtered							OPLS-DA: Minimal[c]								
	Training					Test		Training							Test	
Trial	X[a]	R2Y	Q2	p[b]	Core CV[c]	AUROC	SE	X[a]	R2Y	Q2	p[b]	AUROC(i)	Cancer	Non-cancer	AUROC	SE
A	50	0.451	0.238	4.1×10^{-5}	-	0.67	0.08	24	0.341	0.177	7.8×10^{-4}	0.802	Arabitol5TMS-G<br>Isopropanol-N<br>Mannose-N<br>MatchRI170922-G<br>MyoInositol-N<br>Phenylalanine-C<br>Ribitol5TMS-G<br>Succinate-N<br>Threitol4TMS-G<br>Trehalosealpha-G<br>TwoHydroxyisobutyrat<br>Urea-C	Histidine-C<br>Lysine-N<br>MatchRI129451-G<br>Methionine-C<br>Ornithine-C<br>Proline-C<br>Sorbitol6TMS-G<br>Threonine-C<br>UnmatchedRI16313-G<br>UnmatchedRI22199-G<br>UnmatchedRI24753-G<br>UnmatchedRI28215-G	0.67	0.07
B	62	0.402	0.239	3.7×10^{-7}	-	0.63	0.08	26	0.367	0.231	5.2×10^{-5}	0.839	Glucuronicacid1M-G<br>Mannose-N<br>MyoInositol-N<br>Phenylalanine-C<br>UnmatchedRI10078-G<br>UnmatchedRI10325-G<br>UnmatchedRI11396-G<br>UnmatchedRI18377-G<br>UnmatchedRI18425-G<br>Urea-C	Arginine-N<br>AzelaicAcid2TMS-G<br>Creatine-N<br>Glutamine-C<br>Glycerol-C<br>Histidine-C<br>Lysine-N<br>Methanol-N<br>Ornithine-C<br>Proline-C<br>UnmatchedRI11646-G<br>UnmatchedRI13319-G<br>UnmatchedRI19736-G<br>UnmatchedRI22199-G<br>UnmatchedRI24753-G<br>Xanthine-N	0.66	0.07

[a] Number of metabolites/features in the filtered (t-test p-value <0.3) training set

[b] p-value for CV-ANOVA testing

[c] Minimal list of metabolites determined by multistep iterative process: selection of metabolites contributing most to class difference by multivariate O2PLS analysis controlling for technical and clinical covariates; then iterative OPLS-DA modeling removing metabolites based on their contribution to predictive modeling in test set (YpredPS and T2PS)

Table S3. *Cont.*

	OPLS-DA: Filtered							OPLS-DA: Minimal[c]								
	Training					Test		Training							Test	
**Trial**	**X[a]**	**R2Y**	**Q2**	***p*[b]**	**Core CV[c]**	**AUROC**	**SE**	**X[a]**	**R2Y**	**Q2**	***p*[b]**	**AUROC(i)**	**Cancer**	**Non-cancer**	**AUROC**	**SE**
C	47	0.369	0.219	9.3×10^{-5}	-	0.73	0.06	19	0.311	0.204	1.7×10^{-4}	0.795	Choline-N<br>Erythritol4TMS-G<br>Galactose1MEOX-G<br>Isopropanol-N<br>Mannose-N<br>MyoInositol-N<br>Phenylalanine-C<br>Ribitol5TMS-G<br>Threitol4TMS-G<br>TrimethylamineNoxide<br>UnmatchedRI22127-G	Arginine-N<br>Creatine-N<br>Inosine-N<br>Lysine-N<br>MatchRI201825-G<br>Threonine-C<br>UnmatchedRI19138-G<br>UnmatchedRI19736-G	0.68	0.07
Average	*53*	*0.407*	*0.232*	4.5×10^{-5}		*0.68*	*0.07*	*23*	*0.34*	*0.204*	3.3×10^{-4}	*0.812*			*0.67*	*0.07*

[a] Number of metabolites/features in the filtered (*t*-test *p*-value <0.3) training set

[b] *p*-value for CV-ANOVA testing

[c] Minimal list of metabolites determined by multistep iterative process: selection of metabolites contributing most to class difference by multivariate O2PLS analysis controlling for technical and clinical covariates; then iterative OPLS-DA modeling removing metabolites based on their contribution to predictive modeling in test set (YpredPS and T2PS)
